# Effect of using different strips on reducing the most common error in panoramic imaging: A randomised controlled trial on palatoglossal air space shadow

**DOI:** 10.1002/jmrs.753

**Published:** 2024-02-07

**Authors:** Andisheh Mokhtari, Sedigheh Razi, Kasra Rahimipour, Tahmineh Razi

**Affiliations:** ^1^ Faculty of Dentistry Tabriz University of Medical Sciences Tabriz Iran; ^2^ Department of Oral and Maxillofacial Radiology, Faculty of Dentistry Tabriz University of Medical Sciences Tabriz Iran

**Keywords:** Diagnostic error, palatoglossal airspace error, panoramic radiography, patient positioning, radiographic error

## Abstract

**Introduction:**

Panoramic radiography quality can be impaired by some errors such as positioning errors. Palatoglossal air space shadow error is one of the most common positioning errors and it is due to the tongue not sticking to the roof of the palate. Techniques used to deal with this error might help prevent unnecessary radiation to patients and save them time and money. The study aimed to investigate the effects of using celluloid matrix and edible tapes (fruit leather and chewing gum) on reducing the palatoglossal air space shadow error in panoramic imaging.

**Methods:**

In our study, 270 patients referred to the Department of Radiology were randomised into three groups: a control group, a celluloid matrix group and an edible tapes group. Before panoramic imaging, all patients were instructed to adhere their tongues to the roof of their mouths, with the distinction that for the celluloid matrix and edible tapes groups, patients were asked to place celluloid tapes, fruit leathers, or chewing gums on their tongues before doing so. The routine imaging process was then performed, and the results were compared across groups to evaluate the incidence of palatoglossal air space shadow error.

**Results:**

The number of error‐free images in each fruit leather, chewing gum and celluloid tape group were significantly higher than the control group (all cases *P* < 0.05). The chances of error‐free images in the fruit leather groups were the highest (9.57 times). The age (*P* = 0.136) and gender (*P* = 0.272) of patients had no significant effect on the results of interventions.

**Conclusion:**

The application of fruit leathers, chewing gums and celluloid tapes reduced the palatoglossal air space shadow error of panoramic imaging.

## Graphical abstract

Patients (*n* = 270) were randomised into three groups to investigate the effect of using celluloid matrix and edible tapes (fruit leather and chewing gum) on reducing the palatoglossal air space shadow error in panoramic imaging. Results showed that the number of error‐free images in each group was significantly higher than the control group, with the fruit leather groups having the highest chance of error‐free images (9.57 times). The age and gender had no significant effect on the results of interventions.

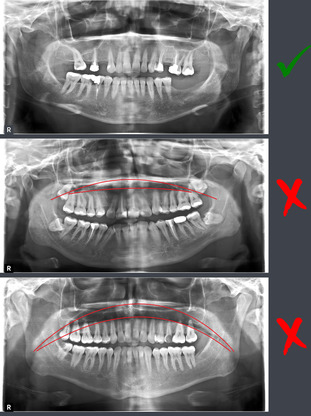

## Introduction

Panoramic radiography (PR) is an extra‐oral imaging technique and is considered an effective, fast and relatively inexpensive diagnostic tool to examine dentofacial structures.^1^, ^2^, ^3^, ^4^ Various errors such as open lips, shifted or tilted head position, inappropriate exposure parameters, movement of the patients, etc., have been reported for panoramic imaging, which reduce the quality of the images. The low quality of the radiographic images can result in misinterpretation by dentists, eventually leading to incorrect treatment plans. In addition, panoramic imaging errors ultimately can cause the repetition of radiography.^5^, ^6^ In addition to wasting time and money, radiography repeating causes excess radiation exposure and increases the risk of potential damage in patients and medical radiation operators, including meningioma, brain tumours and cancers,^7^, ^8^ especially thyroid and leukaemia.^9^, ^10^ Considering that the number of exposures to diagnostic radiation is relatively high during the lifetime, even a small increase related to the risk of cancer is of significant public importance.^11^

Thus, numerous studies have highlighted the necessity of minimising medical and dental diagnostic radiation exposure as much as possible.^12^, ^13^, ^14^, ^15^ Patient positional errors have been identified as the most frequent panoramic imaging errors,^16^, ^17^, ^18^ with some studies suggesting this is the case for nearly 95% of reported errors.^19^ Positional error and patient movement are two common reasons to reject dental imaging including intra‐oral, extra‐oral and cone‐beam computed tomography (CBCT).^20^ Thus, it is suggested that all dental radiography staff receive regular training focused on correct positioning techniques and reduction of patient movement.^20^

The typical patient positioning errors in PR include failure to rest the tongue against the palate, head positioned forward, head positioned backward, head twisted towards the left or right, head tilted to the left or right, the chin too high, the chin too low, a slumped position and inadequate neck extension, not resting the chin on the chin support, failing to use the bite guide, lips opened, patient movement during imaging and not removing the metal objects or prostheses.^21^, ^22^, ^23^ Among all the positional errors mentioned above, the most common error is the failure to keep the tongue against the palate.^24^, ^25^, ^26^

Due to the failure of holding the tongue against the palate, the palatoglossal airspace (PGA) appears in panoramic radiographs as a dark radiolucent band under the hard palate and on the apices of the anterior maxillary teeth.^27^, ^28^ This artefact significantly reduces the quality of the panoramic radiograph as it may be difficult or impossible to interpret the desired periapical region for correct diagnosis and treatment planning (for example, making it difficult to interpret cystic lesions in the anterior area of the upper jaw). In addition, when PGA falls on the ramus of the mandible, it may be mistaken for a fracture.^29^

The PGA may be seen unilaterally and asymmetrically as a localised radiolucent area if the patient swallows during panoramic imaging and momentarily does not touch the roof of the mouth.^30^ If the patient places only the tongue tip behind the maxillary teeth, a larger airspace is formed between the tongue and the hard palate.^26^, ^28^, ^29^

As previous studies suggest, the average incidence of the error of not holding the tongue against the palate is nearly 40%.^31^, ^32^ However, some studies have estimated that the frequency of this error in PR can even reach 80–90%.^26^, ^33^ Also, the tongue not being in contact with the palate is considered the most common error in the mixed dentition period. Overall, the incidence of positional errors, particularly the distance between tongue and palate, is reportedly higher in the mixed dentition period than in the permanent dentition period.^27^, ^33^

The main cause of PGA error is the patient's lack of understanding of the tongue‐palate position and the radiography technicians’ inability to clearly explain this issue to the patient and the lack of effective explanation and communication between the technician and the patient.^26^ During panoramic imaging, patients often cannot hold their tongue in a still position without moving, fully attached to the palate, and the technician cannot check that the patient has followed the instructions and that the tongue is in the correct position.^28^, ^32^, ^33^ Therefore, the operator should allocate sufficient time and use appropriate techniques to position the patient.^19^, ^20^

Although several studies have emphasised the importance and necessity of minimising PGA error, there is little research on the practical methods to achieve this goal.

It seems necessary to minimise the mentioned error using simple and practical techniques. In this regard, the present study evaluates the effect of using celluloid matrix tapes and oral tapes as a simple and cheap method for proper positioning of the tongue on reducing the palatoglossal airway shadow error in panoramic imaging.

## Materials and Methods

This randomised controlled trial was conducted at the Oral and Maxillofacial Radiology Department, Faculty of Dentistry, Tabriz University of Medical Sciences, Iran, from 29 September 2021 to 20 February 2022. The study protocol adhered to the CONSORT guidelines and was approved by the university's ethics committee (Institutional Review Board: IR.TBZMED.REC.1399.730). The trial registration number NCT06149234 at ClinicalTrials.gov was obtained upon the completion of the study.

### Sample size and participants

Since there were no similar studies to refer to, a pilot study was done with 30 patients from the oral and maxillofacial radiology department to figure out a suitable sample size. The patients were randomly divided and assessed in two groups of 15, each testing different materials: celluloid matrix tape and strips of food material. The initial results were promising, with a 99% success rate in the celluloid matrix tape group and 90% in the strips of food material group. Considering a Type I error of 0.05 and study power of 80%, 76 samples for each study group were calculated. To be on the safe side, this number was raised by an additional 20%, meaning 90 samples per group were used in the study. So, in total, the study worked with 270 samples, split evenly into three groups.

### Study design

A total of 270 panoramic radiographs were acquired using a dental X‐ray system (DR Imaging System, RAYSCAN α‐P, South Korea) according to the instructions of the manufacturer at 50/60 Hz, 60–90 kW, 4–17 mA and an acquisition time of 14 s in the Scanner 2.0.1 software. The images were displayed and examined on an LCD (liquid‐crystal display) monitor (Dell E2014HF) with dimensions of 14.1 × 6.5 × 18.7 in. and a pixel pitch of 0.27 mm.

In this study, all panoramic images were taken by a technician according to the patient positioning standards. The exposure settings were selected based on the patient's size and weight.

The main inclusion criterion was age above 15 years. Patients with a history of trauma, lesion, swelling, disseminated infection, physical and mental disabilities, severe gag reflex, cleft palate, difficulty in speech and tongue movement, allergy to the materials used in this study, and those reluctant to cooperate were excluded from the study.

**Figure 1:**
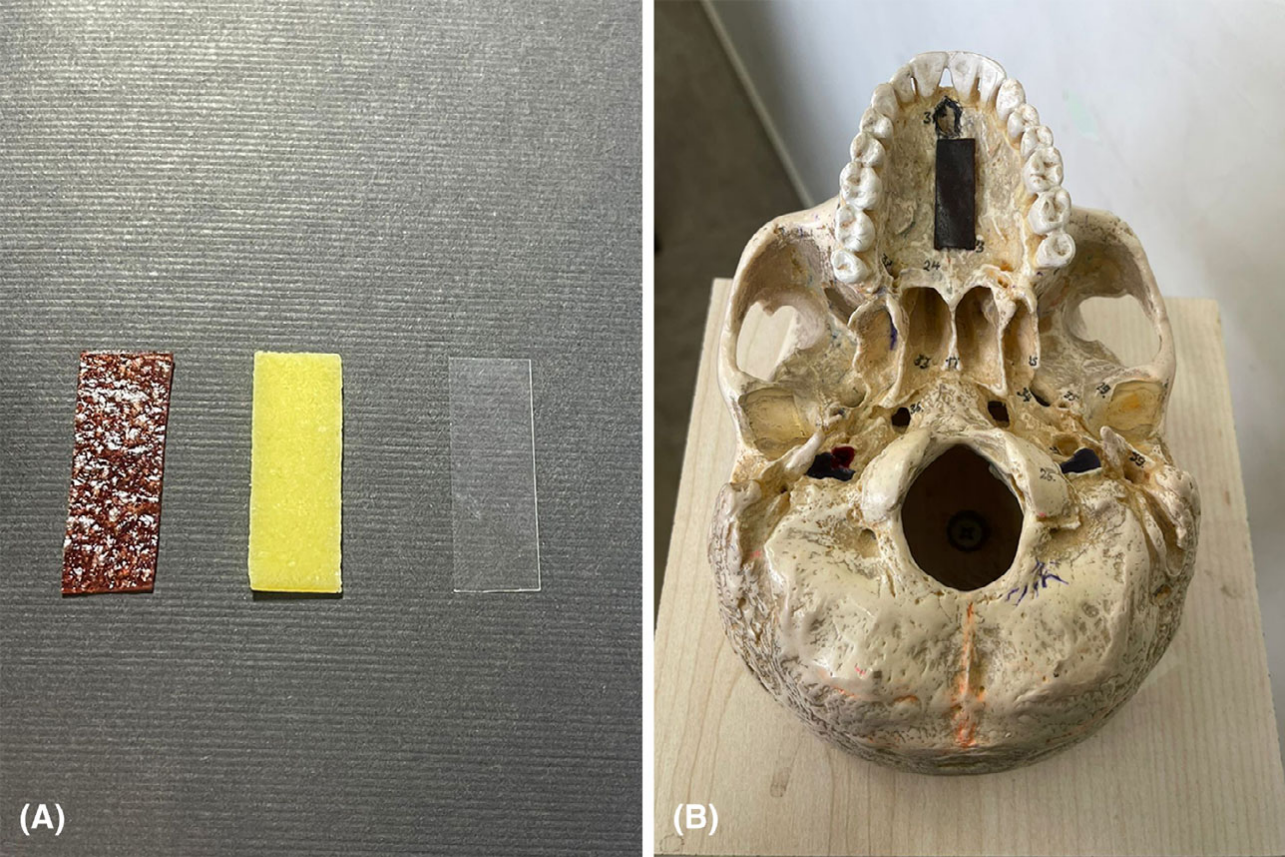
(A) From left to right an example of sour fruit leather (thickness: 1 mm), chewing gum (thickness: 1 mm) and celluloid matrix strip (thickness: 5 μm). (B) Approximate positioning of the strips.

The patients were randomised into three groups. Ninety patients received celluloid matrix strips of transparent polyester (Maquira Dental Products, Maringá, Brazil) with a 10 mm width and 30 mm length. In the second group, 90 patients were given edible strips. They were divided into two subgroups of 45 people. Forty‐five patients received 10 × 30 mm strips of either commercial chewing gum (Banana Orion) with sweet banana flavour and 45 patients received sour fruit leather. Finally, as the third group, 90 patients were selected as the control group without giving any specific instructions. Figure 1 shows the different strips used in this study and the approximate positioning of the strips at the palate of the patients.

### Instruction of patients

Panoramic imaging was carried out in the control group without any intervention (except for as a routine procedure, all of the patients in the control group were instructed to stick their tongue to the palate) and in accordance with the standard protocols of the Department of Oral and Maxillofacial Radiology.

Before panoramic imaging, the patients in edible and celluloid strip groups were instructed to put the given strips on their tongues. The strips were positioned in the anterior–posterior dimension at the middle third of the tongue, and in the transverse dimension in such a way that the centerline of the strip was placed in the central groove of the tongue (Fig. 1B). When adjusting the final head position in the machine, they were asked to elevate the strip to the roof of the mouth and hold it against the palate during the imaging procedure.

### Assessment of radiographs

**Figure 2:**
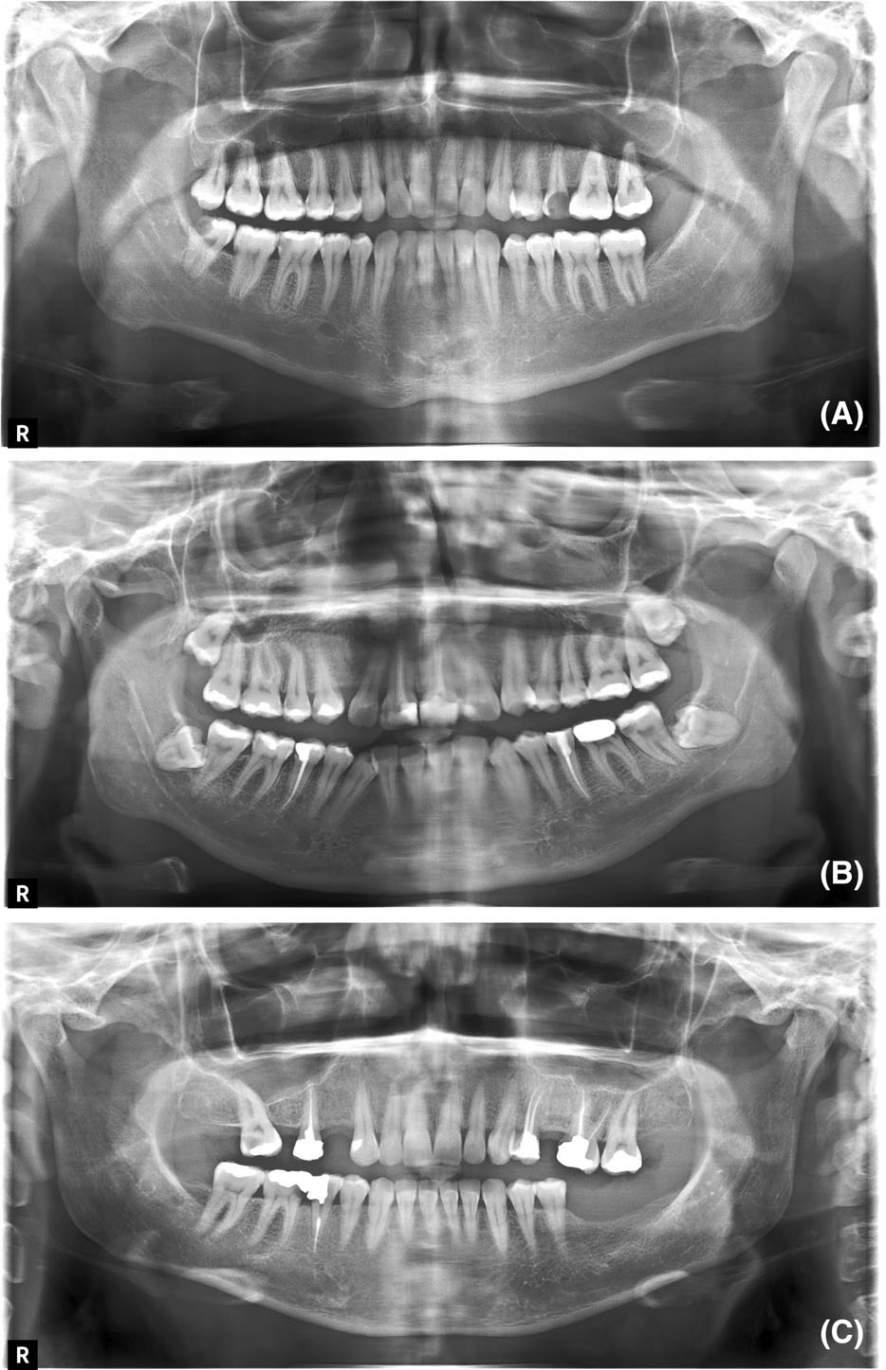
Examples of radiographic images: (A) An unacceptable radiographic image classified in this study based on Palatoglossal air space shadow error which can obscure critical anatomical details, potentially leading to misdiagnosis or the need for additional imaging procedures. (B) An acceptable radiographic image classified in this study based on Palatoglossal air space shadow error. (C) An excellent radiographic image classified in this study based on Palatoglossal air space shadow error.

An oral and maxillofacial radiologist assessed the radiographs in single‐blinded random selection to identify and record the errors. The radiographs were classified as diagnostically acceptable, unacceptable and excellent based on the degree of the palatoglossal air space shadow error (Fig. 2).

### Randomisation and blinding

**Figure 3:**
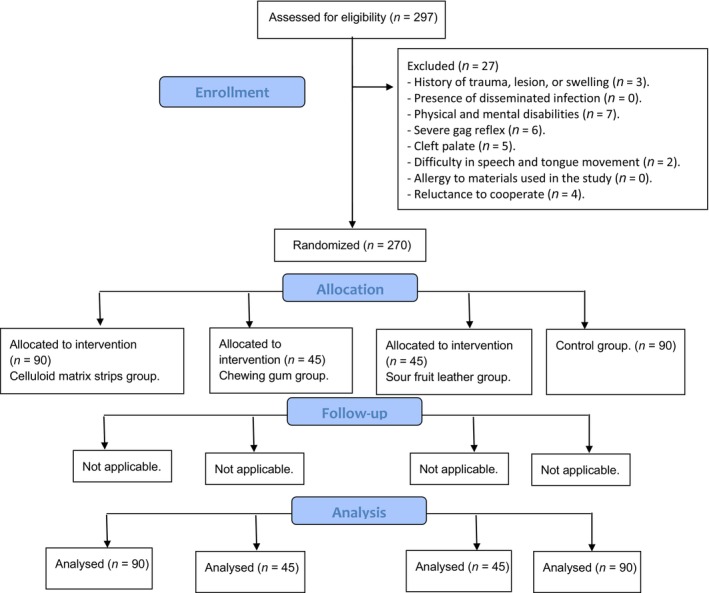
CONSORT flow diagram of the study.

Participants were randomly assigned into three groups using the randomiser feature of SPSS software version 26 (IBM Corp., New York, NY, USA; formerly SPSS Inc., Chicago, IL, USA). The groups were: a control group, a celluloid matrix group and an edible tape group. The recruitment process and participant flow are detailed in the Consort Flow Diagram (Fig. 3). To minimise bias and ensure the validity of results, the radiologist assessing the radiographs was blinded to the group assignments.

A prepared box containing 270 papers, each marked with a number from 1 to 270, was utilised for the study's randomisation process. Each paper indicated which group, intervention or control, the number corresponded to. This box was entrusted to the technician responsible for panoramic imaging. An undergraduate student, who was the executor of this study, was involved in the randomisation process.

Patients who were referred to the Department of Oral and Maxillofacial Radiology for panoramic imaging, either for diagnostic or therapeutic purposes, were considered for inclusion in the study. If deemed eligible and consenting to participate in the study, they were randomly assigned a number upon arrival. This number was generated by the student using SPSS software and then handed to the patient. The technician, based on this number, selected the corresponding paper from the box to determine the group allocation for the patient. Necessary instructions were then provided to the patient before the imaging was conducted.

The radiologist interpreting the images was blinded to this box and the group associated with each number. The radiologist recorded the interpretation of the panoramic images (categorised as diagnostically acceptable, unacceptable, or excellent) against the image number. Subsequently, this data was collected by the student for statistical analysis.

### Statistical analyses

Statistical analyses were carried out using SPSS software. The qualitative data were represented as frequency (percentage), and normal quantitative data were expressed as mean ± SD. The Chi‐square test was used to compare the qualitative variables, and one‐way analysis of variance (ANOVA) was used for quantitative variables with normal distribution. The intervention outcomes were evaluated using a logistic regression model. The differences with *P* < 0.05 were considered statistically significant for all tests.

## Results

Of the 270 included patients in this study, the imaging results in terms of PGA error were reported to be excellent in 155 (57.4%), diagnostically acceptable in 56 (20.7%) and unacceptable in 59 (21.9%) cases.

A Chi‐square test found no statistically significant relationship between gender and imaging results in terms of PGA error (*P* = 0.157). Similarly, analysis by one‐way ANOVA indicated no statistically significant association between patients’ age and imaging results in terms of the PGA error (*P* = 0.125).

The Chi‐square analysis revealed a statistically significant relationship between the strip material used for tongue positioning and the radiographic result (*P* < 0.0001). Frequency distribution of the radiography results based on the strip materials showed that the lowest frequency of the unacceptable images was found in the edible strip (fruit leather) group. At the same time, the highest frequency of the unacceptable images was related to the control group. The highest and lowest rate of acceptable images (excellent and/or diagnostically acceptable) were associated with the edible strip (chewing gum and fruit leather) and control groups, respectively (Table 1).

**Table 1:** Imaging results based on the strip materials used for tongue positioning.

Group	Acceptance			*P*‐value
	Excellent	Diagnostically acceptable	Unacceptable	
Edible strips – fruit leather	34 (75.5%)	8 (17.7%)	3 (6.6%)	>0.001
Edible strips – chewing gum	34 (75.5%)	6 (13.3%)	5 (11.1%)	
Celluloid matrix strips	54 (60%)	24 (26.6%)	12 (13.3%)	
Control	33 (36.6%)	18 (20%)	39 (43.3%)	

Data are shown as frequency (percentage).

Table 2 shows the distribution of age and gender among three groups with sub‐groups when applicable for Group 2. In terms of ages as given by the range, mean and standard deviation, there is a relatively balanced distribution in all groups and sub‐groups. The distribution on gender was varied but did not differ much between groups and among sub‐groups since *P*‐value = 0.763. This validates that differences in age and gender did not greatly affect the results of this study.

**Table 2:** Characteristics of study participants.

Characteristic	Group 1 (*n* = 90)	Group 2a (*n* = 45)	Group 2b (*n* = 45)	Group 3 (*n* = 90)	*P*‐value
Age (years)	36.9 ± 13.6	39.8 ± 15.7	31.3 ± 11.9	36.6 ± 14.6	0.036
Gender					
Male	48 (53.3%)	21 (46.7%)	12 (26.7%)	37 (41.1%)	0.028
Female	42 (46.7%)	24 (53.3%)	33 (73.3%)	53 (58.9%)	

Values for age are presented as range (mean ± SD). Values for gender are presented as count (percentage). Group 1, celluloid matrix strips group; Group 2a, chewing gum group; Group 2b, sour fruit leather group; Group 3, control group.

For the logistic regression analysis, the images were divided into acceptable (excellent and/or diagnostically acceptable) and rejected groups, and the effect of the strip materials on the imaging outcome was measured after controlling for age and gender. The effect of age on the imaging results was insignificant (*P* = 0.136). The chance of radiograph acceptance in women was 0.69‐fold higher than in men; however, this difference was not statistically significant (CI = 0.36–1.33, OR = 0.69, *P*‐value = 0.27; Table 3).

**Table 3:** Imaging results based on the logistic regression analysis and the effect of the strip materials after controlling for age and gender.

	OR (95% CI)	*P*‐value
Age	0.98 (0.96–1.01)	0.136
Gender		
Male	Reference	
Female	0.69 (0.36–1.33)	0.272
Strip material		
Control	Reference	
Edible strips – fruit leather	9.57 (2.74–33.45)	>0.001
Edible strips – chewing gum	6.9 (2.44–19.53)	
Celluloid matrix strips	5.36 (2.53–11.37)	

CI, confidence interval; OR, odds ratio.

The strip materials used for tongue positioning significantly affected the imaging outcome. The chance of imaging acceptance in patients using fruit leather was 9.57 times higher than in the control group (*P* < 0.001). Patients who received chewing gum had a 6.9 times higher chance of radiograph acceptance than the control group (*P* < 0.001). Finally, the chance of having acceptable radiographs in the celluloid strip group was 5.36 times higher than in the control group (*P* < 0.001).

## Discussion

PR is one of the main diagnostic aids in dentistry which is used in various situations, including dental caries, tooth fractures, bone pathology, temporomandibular joint disorders, periodontal diseases and oral surgery.^1^, ^34^, ^35^ However, radiographic interpretation may be impaired if errors occur during the imaging process.^36^

Patient positioning during imaging is a major challenge in PR.^37^, ^38^ Positional errors that cannot be seen by the technician, such as the PGA error, are considered among the most challenging errors because they depend a lot on the patient's cooperation.

A strategy for sticking the tongue to the palate is to ask the patient to make a clicking sound or swallow during the radiography, but considering the need for the patient's understanding and cooperation, it seems not to be an entirely sufficient method.^39^, ^40^

The present study suggests that the material type used for tongue positioning significantly influences the rate of error‐free radiographs. The frequency of error‐free images in groups that received fruit leather, chewing gum, or celluloid strips was significantly higher than in the control group. Although all three evaluated materials effectively reduced the incidence of PGA shadow error, the highest and lowest chance of error‐free images compared to the control group was obtained in the fruit leather and celluloid strip groups, respectively. These findings indicate that although celluloid strips offer various properties, including high tensile strength, flexibility and transparency.^41^, ^42^ they provide lower stickiness than fruit leather and gum to hold the tongue against the palate. Edible materials seem more favourable for the patients, and the fruit leather, due to its sourness, had a higher effect on keeping the tongue against the palate. In addition, the opacity of fruit leather and chewing gum compared to celluloid appears to positively influence radiograph quality regarding the incidence of PGA shadow error.

Out of 270 panoramic radiographs reviewed in this study, 59 radiographs (21.9%) showed PGA error. Radiographs with PGA error were observed in 25.4% of male patients and 19.1% of female patients. However, the difference was not reported to be statistically significant, the lower occurrence of this error in women may be due to the greater compliance of women with the requests of the medical staff in the field of diagnosis and treatment and their greater attention to details.

Bagherpour et al.^33^ reported a significantly higher incidence of patient positioning error in men than in women, in contrary to the results of our study. A possible explanation for this discrepancy is that they evaluated the overall incidence of positional errors in panoramic imaging between men and women, but the focus of our study was only PGA error.

On the other hand, Bagherpour et al.,^33^ could not find a statistically significant difference between patients’ age and imaging results, similar to the results of our study. One of the limitations of the present study is that the age group under investigation was over 15 years old, so it is recommended for the future studies to investigate the effect of the proposed method on the age group under 15 years old.^33^

A well‐known attempt to reduce the incidence of PGA in PR was made in the study of Cordesmeyer et al.^40^ in which a membrane funnel protector (MFS) was used to fix the tongue in the proper position. However, the proposed method was expensive and required an intraoral device and detailed instructions on tongue position, and the use of an intraoral device may be inappropriate for patients with intellectual disabilities. Other problems with this method include the need for patient compliance and technician training. In contrast, the oral and celluloid tapes introduced in our study are remarkably inexpensive, simple and widely applicable. In addition, it is much easier for the patient to understand how to use these strips.^40^

Another attempt at reducing the PGA is the study by Scott et al., in which the authors proposed a simple respiration control method to decrease airway shadow in paediatric and disabled patients.^43^ However, this method is technically sensitive. If breathing control instructions are given too early, the tongue will not remain in the correct position during the imaging period, and if instructions are given too late and the patient swallows during the procedure, incomplete PGA and movement of the hyoid bone will be visible. Our proposed method does not have a special technical sensitivity and can be easily trained and used.

In many RCTs, a significant gap usually exists between randomisation and intervention, often leading to participant dropout in later stages.^44^ However, our study's methodology featured a very short interval between patient allocation and imaging. Due to this minimal time gap and the lack of necessity for patient follow‐up, our study experienced no participant dropout, ensuring efficient completion and maintaining the integrity of our research.

## Conclusion

This randomised controlled trial suggested that celluloid matrix and edible strips reduced PGA shadow error in panoramic imaging. The highest and lowest chance of error‐free radiographs compared to the control group were recorded for fruit leather and celluloid strips, respectively. Thus, chewing gum, celluloid strip and especially fruit leather, a cheap and accessible foodstuff, can help minimise the error of not placing the tongue against the palate during panoramic imaging. Further research is suggested to develop similar efficient strategies to achieve this goal.

## Conflict of Interest

The authors declare no conflict of interest.

## Data Availability

The data that support the findings of this study are available from the corresponding author upon reasonable request.
